# Pathogenesis and symptom of early hemorrhage in extrafollicular ovarian pregnancy onset at 4weeks gestation: A case report

**DOI:** 10.1016/j.ijscr.2025.111647

**Published:** 2025-07-10

**Authors:** Kuniaki Ota, Toshifumi Takahashi, Yoshiaki Ota, Wataru Saito, Hirotake Nishimura, Takuya Moriya, Koichiro Shimoya

**Affiliations:** a Department of Obstetrics and Gynecology, Kawasaki Medical School, Okayama 701-0192, Japan; b Fukushima Medical Center for Children and Women, Fukushima Medical University, Fukushima 960-1295, Japan; c Department of Pathology, Kawasaki Medical School, Okayama 701-0192, Japan

**Keywords:** Ectopic pregnancy, Ovarian pregnancy, Extrafollicular ovarian pregnancy, Intrafollicular ovarian pregnancy, Laparoscopy, Extravillous trophoblast

## Abstract

**Introduction:**

Ovarian pregnancy is a rare form of ectopic pregnancy, accounting for approximately 3 % of cases, with an incidence ranging from 1 in 2100 to 1 in 7000 pregnancies. Its diagnosis is challenging due to nonspecific symptoms and difficulty distinguishing it from corpus luteum cysts or tubal pregnancies. Delayed recognition can lead to life-threatening hemorrhage.

**Presentation of case:**

A 34-year-old gravida 3 para 3 Japanese woman presented with acute abdominal pain and hypovolemic shock 33 days after her last menstrual period. Imaging revealed a right ovarian cystic mass, intra-abdominal bleeding, and an empty uterus. Emergency laparoscopy identified a 5-mm gestational sac-like lesion on the right ovary. Laparoscopic wedge resection was performed. Pathological analysis confirmed extravillous trophoblast invasion into ovarian stromal vessels. A corpus luteum was observed at a separate location, supporting the diagnosis of secondary extrafollicular ovarian pregnancy. The patient recovered uneventfully.

**Discussion:**

Ovarian pregnancies are classified as primary or secondary, and intrafollicular or extrafollicular. This case demonstrated secondary extrafollicular implantation with vascular invasion. Updated diagnostic criteria emphasize intact fallopian tubes, hemorrhagic ovarian lesions, and pregnancy tissue identification. Early detection remains difficult, particularly before 5 weeks gestation, and diagnosis often requires surgical and pathological confirmation.

**Conclusion:**

This case underscores the importance of early recognition and laparoscopic management of ovarian pregnancy. Pathological findings aid in understanding implantation mechanisms and differentiating from similar adnexal conditions. Minimally invasive surgery enabled successful hemostasis and fertility preservation. Improved clinical awareness is essential to reduce complications associated with this rare condition.

## Highlights

• Ovarian pregnancy is rare and has ambiguous clinical and imaging criteria.
• A 34-year-old woman was diagnosed with an actively bleeding ovarian pregnancy.
• Following wedge resection, she recovered without complications.
• Surgical intervention is crucial in ovarian pregnancy management.

## Graphical abstract

**Figure:**
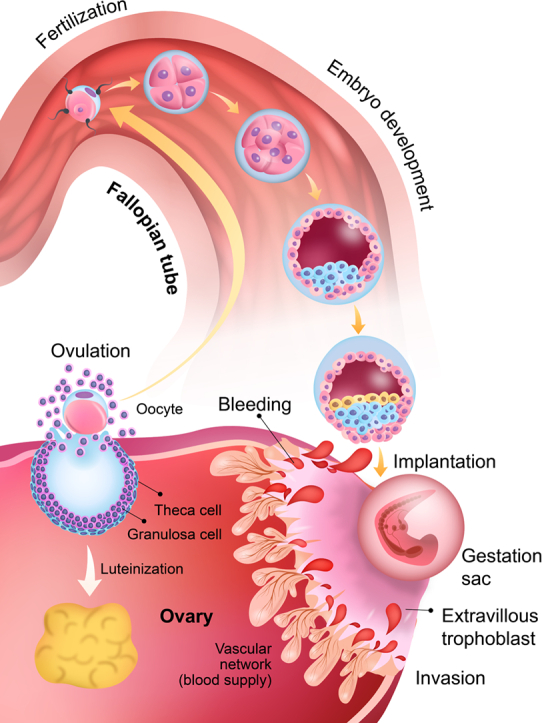
Proposed Mechanism: Extrafollicular Ovarian Pregnancy from Tubal Fertilization to Ovarian Implantation.

## 1 Introduction

Ovarian pregnancy is a rare condition, occurring in approximately 1/2100 to 1/7000 pregnancies overall and in 3 % of ectopic pregnancies [1]. Owing to its unclear pathophysiology and ambiguous diagnostic criteria, it can be difficult to identify, and delayed diagnosis can be life-threatening [2].

Between 2011 and 2024, 85 case reports involving ovarian pregnancy were published internationally [[3], [4], [5]]. Most of these pregnancies were typically discovered during the first trimester, with progression beyond 8 weeks being exceedingly rare [3]. Given the infrequency of its occurrence, sharing clinical experiences is crucial, as misdiagnosis can result in fatal outcomes, whereas overdiagnosis may lead to the unnecessary loss of a woman's fallopian tubes or ovaries [6]. Ovarian pregnancies are broadly categorized as primary or secondary. Primary ovarian pregnancy typically results from ovulatory disturbances, allowing fertilization to occur within the ovarian follicle itself. In contrast, secondary ovarian pregnancy arises when a tubal abortion or rupture leads to implantation of the conceptus in the ovarian stroma. Furthermore, ovarian pregnancies are subtyped into intrafollicular and extrafollicular forms. While intrafollicular pregnancies are generally of primary origin, extrafollicular cases may be associated with either primary or secondary mechanisms [7].

In this case report, we describe a 34-year-old woman who presented with acute abdominal pain 33 days after her last menstrual period, raising the suspicion of an ectopic pregnancy. Emergency laparoscopy was performed, which suggested a rare right ovarian pregnancy that was subsequently diagnosed histopathologically as right extrafollicular ovarian pregnancy. This case report provides valuable insights into the pathogenesis of ovarian pregnancy and highlights the importance of prompt recognition and early intervention to optimize clinical outcomes. This case is reported in accordance with Revised Surgical CAse REport (SCARE) guideline [8].

## 2 Case presentation

A Japanese women in her early thirties (gravida 3, para 3), which had no history of infertility treatment or ectopic pregnancy, was admitted in emergency ward after presenting with acute abdominal pain 33 days after her last menstrual period, with a normal menstrual cycle history. Upon examination, she appeared pale and exhibited hemodynamic instability, with a pulse rate of 100 bpm and a systolic/diastolic blood pressure of 60/45 mmHg (shock index = 1.7). Laboratory tests revealed the presence of anemia (based on a hemoglobin concentration of 9.0 g/dL) and a β-human chorionic gonadotropin (β-hCG) level of 2054.1 IU/mL.

**Fig. 1:**
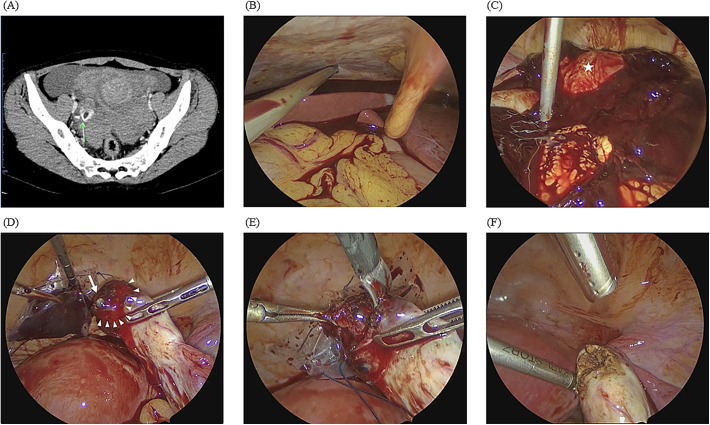
(A) Axial contrast-enhanced computed tomography scan through the pelvis showing bleeding from the right ovary (green arrow). Exploratory laparoscopic imaging reveals a huge amount of bleeding in the abdominal cavity, especially extending to the liver, and with the uterus visible within the hematoma (star). (D) Laparoscopic imaging showing the presence of the gestational sac on the surface of the ovary (arrow), with persistent bleeding from the ovary over a wide area (arrowhead). (E) Laparoscopic imaging of the wedge resection of the pregnancy tissue using forceps. (F) Laparoscopic imaging of the electrocoagulation of the surface of the excision site to prevent residual pregnancy tissue and the development of gestational trophoblast disease. (For interpretation of the references to color in this figure legend, the reader is referred to the web version of this article.)

Urgent transvaginal ultrasonography revealed an empty uterus, a substantial amount of free fluid, and the presence of blood clots in the Douglas and Morrison’s pouches. Furthermore, the left adnexal region was obscured by clots, whereas the right adnexal region exhibited a 9-cm cystic mass with a hyperechoic decidual reaction, strongly suggesting an ectopic pregnancy at 4 weeks and 5 days of gestation. Additionally, an echo-free space surrounding the liver was indicative of massive intra-abdominal hemorrhaging. Pelvic contrast-enhanced computed tomography was subsequently performed, and contrast medium extravasation confirmed active bleeding from the right ovary (Fig. 1A).

Emergency laparoscopy was performed, revealing a hemoperitoneum of approximately 1000 mL in volume extending to the liver (Fig. 1B and C). The uterus was normal in size, both fallopian tubes appeared smooth and intact, and the left ovary was unremarkable. However, a 5.0-mm gestational sac-like mass was identified on the surface of the injured, actively bleeding right ovary (Fig. 1D). Laparoscopic wedge resection of the affected ovarian tissue, including the gestational sac-like mass, was performed (Fig. 1E). Hemostasis was achieved using bipolar scissors, and the affected ovary was repaired (Fig. 1F). No apparent corpus luteum was observed laparoscopically in either ovary.

**Fig. 2:**
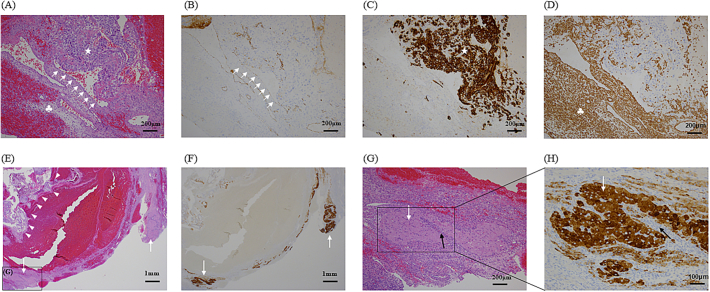
(A) Microscopic image of hematoxylin-eosin staining demonstrating extravillous trophoblast invasion (white star) into the blood vessels (arrow) of the ovarian stroma (clover) of resected tissue. Scale bar: 200 μm. (B) Immunohistochemical labelling of CD31 (arrows) for detection of the vascular endothelium of the affected ovary Scale bar: 200 μm. (C) Immunohistochemical labelling of cytokeratin (AE1/AE3) (white star) for the detection of extravillous trophoblasts. Scale bar: 200 μm. (D) Immunohistochemical labelling of vimentin for detection of the ovarian stroma (clover). Scale bar: 200 μm. (E) Hematoxylin-eosin staining demonstrating extravillous cytotrophoblasts in cell islands (arrowhead) invading the ovarian stroma with a corpus luteum (arrow). Scale bar: 1 mm. (F) Immunohistochemical labelling of inhibin for detection of the corpus luteum (arrows). Scale bar: 1 mm. (G, H) In the hematoxylin-eosin staining, the corpus luteum, detected using immunohistochemical labelling of inhibin, was composed of the granulosa-lutein (white arrows) and theca-lutein cells (black arrows). Scale bars: 200 and 100 μm, respectively.

Postoperatively, the patient received two units of packed red blood cells. Her recovery was uneventful, and her β-hCG levels dropped to 480.1 mIU/mL after 2 days. She experienced no postoperative complications and was discharged on the 4th day postoperatively. Pathological analyses provided insights into the vascular disruption mechanism in ruptured ovarian pregnancy. Hematoxylin-eosin staining revealed collapsed ovarian vascular networks, indicating that the bleeding was caused by extravillous trophoblast invasion into the blood vessels of the ovarian stroma (Fig. 2A). Anti-CD31 antibody labelling, a marker of vascular endothelium, confirmed that the invaded structures were indeed blood vessels (Fig. 2B), whereas the extravillous trophoblasts and ovarian stroma were positively labelled with anti-cytokeratin (AE1/AE3) and anti-vimentin antibodies, respectively (Fig. 2C, D). Hematoxylin-eosin staining identified a corpus luteum composed of granulosa- and theca-lutein cells at separate location from the trophoblast invasion site (Fig. 2E), and anti-inhibin antibody labelling confirmed its functional status as an endocrine gland (Fig. 2F–H). Collectively, these findings suggested that ovulation had occurred ipsilaterally and that the rupture resulted from trophoblast-induced vascular invasion and network collapse at another site of the ovulated follicle.

## 3 Discussion

While ovarian pregnancies remain challenging to identify, the diagnostic criteria have evolved alongside advancements in imaging technology and laparoscopic techniques. In 1878, Spiegelberg [9] first established criteria for the surgico-pathological diagnosis of primary ovarian pregnancy, which required the presence of intact fallopian tubes separate from the ovary, gestational sac implantation into the ovary, and evidence of ovarian tissue attachment to the pregnancy specimen. Although these criteria are still cited, their relevance is limited in modern practice because they were established based on autopsy findings. With the advent of laparoscopic techniques, Ren et al. proposed updated diagnostic criteria, which included the presence of intact fallopian tubes, hemorrhagic lesions on the ovarian surface, and visible or microscopic pregnancy tissue within the pelvic cavity [4]. However, distinguishing between ovarian pregnancy with a blood clot from a corpus luteum rupture and tubal pregnancy miscarriage remains challenging, making pathological confirmation essential. In this patient, the gestational sac on the ovarian surface allowed for the direct visualization of the pregnancy tissue, which is extremely rare and fully aligns with the criteria proposed by Ren et al. [4].

**Fig. 3:**
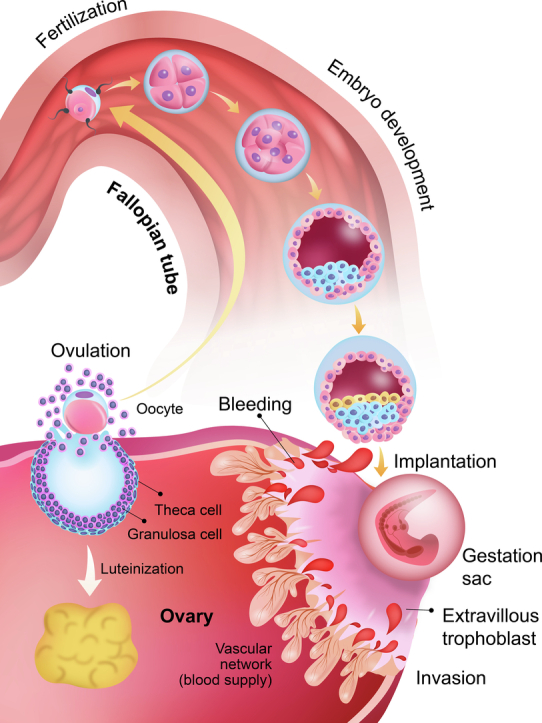
A diagram depicting the proposed mechanism underlying this case of extrafollicular ovarian pregnancy. It is speculated that oocyte ovulation in the right ovary and embryonic development and backflow from the fallopian tube resulted in invasion of the ipsilateral ovary.

Notably, preoperative diagnosis of ovarian pregnancy is challenging [10]. Typically, clinicians begin monitoring pregnancies at 5 weeks of gestation to confirm the presence of a gestational sac within the uterus [11] however, in this case, the embryo likely implanted in the ovary before a gestational sac could be detected in the uterus. The chorionic villi had invaded the ovarian tissue, leading to bleeding at approximately 5 weeks of gestation. Ovarian pregnancies are thought to rupture earlier than other ectopic pregnancy types because of the thin, inelastic surface of the cortex of ovarian tissue. This case provides valuable pathological and visual insights into the mechanisms underlying ovarian pregnancy, and its pathophysiology and clinical presentation. In 1968, Tan and Yeo [12] classified ovarian pregnancies into intrafollicular and extrafollicular types based on the underlying etiology. In the intrafollicular type, the oocyte remains within the follicle, where sperm enters through the ruptured follicle to fertilize it. Conversely, in the extrafollicular type, the oocyte is released but implants on the ovarian surface post-fertilization [12]. In intrafollicular pregnancies, some have speculated that rupture may occur when the gestational sac remaining within the follicle undergoes expansion beyond the follicle's capacity, whereas in extrafollicular pregnancies, villous trophoblasts may invade the ovarian cortex and stroma, leading to tissue destruction and hemorrhage owing to the absence of maternal decidua in the ovary. According to the classification of Tan and Yeo, this case could be classified as an intrafollicular pregnancy because of the presence of a corpus luteum in the ipsilateral ovary. However, we believe that the intrafollicular environment is unsuitable from the time of fertilization to the blastocyst development. Therefore, we assumed that the embryo, which was fertilized and developed in the right fallopian tube, flowed back and became implanted in the right ovary. The histopathological analysis confirmed that the follicle did not rupture; instead, extravillous trophoblasts penetrated the blood vessels in the ovarian stroma, causing bleeding. Furthermore, the embryo may have undergone implantation into the ovary from the outside, as the corpus luteum was located separately from the extravillous trophoblast invasion site (Fig. 3).

Ovarian pregnancies often present as emergencies owing to the abdominal pain they induce, leaving little time for preoperative evaluation. Ultrasonography remains the primary diagnostic imaging technique because of its accessibility. Similar to other ectopic pregnancies, ovarian pregnancy is diagnosed when an extrauterine gestational sac with a yolk sac is detected, specifically on an ovary. However, yolk sacs or embryos are rarely observed, and ultrasound findings can be ambiguous, often resembling a corpus luteum, which is a physiological post-ovulatory structure [1]. On Doppler ultrasound, a “ring of fire” pattern—characterized by increased peripheral vascularity surrounding a gestational sac—is often observed in tubal ectopic pregnancies. However, this finding is not specific for ovarian ectopic pregnancy, as corpus luteal cysts can exhibit similar vascular flow patterns, limiting its diagnostic utility in such cases [[13], [14], [15]]. A recent study reported that fetal heartbeats were easier to detect in ovarian than in tubal pregnancies when skilled operators used advanced techniques such as color Doppler and pelvic palpation with an ultrasound probe [10]. However, in this case, the patient was only 4 weeks pregnant, and bleeding had already begun, making it nearly impossible even for an experienced operator to detect a fetal heartbeat. While modern ultrasound machines offer improved resolution, detecting a gestational sac or fetal heartbeat before 5 weeks of pregnancy remains challenging. While diagnosing early-stage ovarian pregnancy is difficult, in this case, the gestational sac was successfully confirmed laparoscopically.

The greater blood loss in ovarian as compared to tubal pregnancies is attributed to the high ovarian vascularity [10]. Hemodynamically stable patients are increasingly managed laparoscopically, with a recent shift away from laparotomy [16,17]. Ovarian wedge resection is the preferred approach, especially for patients wishing to preserve fertility, whereas oophorectomy is reserved for those with uncontrollable bleeding [4,10]. In this case, laparoscopic ovarian wedge resection was performed, with electrocoagulation of the resection site to mitigate the need for secondary surgery or treatment caused by residual tissue, such as gestational trophoblastic disease. Pathological analysis confirmed complete removal of invasive chorionic tissue.

## 4 Conclusions

In conclusion, this case report describes the rupture of an ovarian pregnancy and confirms the pathological mechanisms underlying the associated bleeding, specifically extravillous trophoblastic invasion into ovarian stromal vessels. Ovarian pregnancy, though rare, can result in massive hemorrhage and life-threatening complications due to the ovary's rich vascularity and limited tissue elasticity. Consequently, laparoscopic wedge resection successfully preserved ovarian function. Histopathological findings, including the presence of a corpus luteum at an ipsilateral site from the trophoblastic invasion, supported the diagnosis of an extrafollicular ovarian pregnancy of secondary origin—likely resulting from retrograde implantation following fertilization in the fallopian tube. Accurate classification, aided by laparoscopic visualization and detailed histopathology, is essential not only for understanding pathogenesis but also for guiding appropriate surgical management. This case underscores the value of interdisciplinary approaches combining imaging, surgery, and pathology in distinguishing ovarian pregnancy from corpus luteal and other adnexal pathologies, and optimizing outcomes in reproductive-age women.

## CRediT authorship contribution statement

KO and TT designed and conceived this case report. KO and TT drafted the manuscript. YO and WS collected data. YO, WS and HN analyzed and interpreted the results. TM and KS supervised this case report. All authors read and approved the final manuscript.

## Informed consent

Written informed consent was obtained from the patient for publication of this case report and accompanying images. A copy of the written consent is available for review by the Editor-in-Chief of this journal on request.

## Ethical approval

The study was conducted according to the guidelines of the Declaration of Helsinki.

## Ethical approval

This study was reviewed and approved by the Human Research Ethics Committee of Kawasaki Medical School (trial registration no.: 5043–03).

## Guarantor

Yoshiaki Ota, M.D., Ph.D. is the guarantor.

## Registration of Research Studies

1.Name of the registry:

NA

2.Unique identifying number or registration ID:

NA

3.Hyperlink to your specific registration (must be publicly accessible and will be checked):

NA

## Source of funding

This research received no external funding.

## Declaration of competing interest

The authors declare no conflicts of interest.
